# Investigating *in vivo* force and work production of rat medial gastrocnemius at varying locomotor speeds using a muscle avatar

**DOI:** 10.1242/jeb.248177

**Published:** 2024-11-13

**Authors:** Caitlin Bemis, Nicolai Konow, Monica A. Daley, Kiisa Nishikawa

**Affiliations:** ^1^ Department of Biological Sciences, Northern Arizona University, Flagstaff, AZ 86011, USA; ^2^ Department of Ecology and Evolutionary Biology, University of California, Irvine, Irvine, CA 92697, USA; ^3^ Department of Biology Studies, University of Massachusetts at Lowell, Lowell, MA 01854, USA

**Keywords:** Rat medial gastrocnemius, Mouse extensor digitorum longus, Muscle mechanics, Fascicle strain, Dynamic muscle response, Work loop

## Abstract

Traditional work loop studies, that use sinusoidal length trajectories with constant frequencies, lack the complexities of *in vivo* muscle mechanics observed in modern studies. This study refines methodology of the ‘avatar’ method (a modified work loop) to infer *in vivo* muscle mechanics using *ex vivo* experiments with mouse extensor digitorum longus (EDL) muscles. The ‘avatar’ method involves using EDL muscles to replicate *in vivo* time-varying force, as demonstrated by previous studies focusing on guinea fowl lateral gastrocnemius (LG). The present study extends this method by using *in vivo* length trajectories and electromyographic activity from rat medial gastrocnemius (MG) during various gaits on a treadmill. Methodological enhancements from previous work, including adjusted stimulation protocols and systematic variation of starting length, improved predictions of *in vivo* time-varying force production (*R*^2^=0.80–0.96). The study confirms there is a significant influence of length, stimulation and their interaction on work loop variables (peak force, length at peak force, highest and average shortening velocity, and maximum and minimum active velocity), highlighting the importance of these interactions when muscles produce *in vivo* forces. We also investigated the limitations of traditional work loops in capturing muscle dynamics in legged locomotion (*R*^2^=0.01–0.71). While *in vivo* length trajectories enhanced force prediction, accurately predicting work per cycle remained challenging. Overall, the study emphasizes the utility of the ‘avatar’ method in elucidating dynamic muscle mechanics and highlights areas for further investigation to refine its application in understanding *in vivo* muscle function.

## Teaser

**Summary:** *In vivo* strain is a more important determinant of force and work production at faster operating frequencies than timing of activation during *ex vivo* work loop experiments.

## INTRODUCTION

Muscles are the primary actuators of animal movements. Fundamental knowledge of muscle structure, function, dynamics and evolution is critical for understanding movement across organizational scales (Schaeffer and Lindstedt, 2013). However, understanding intrinsic muscle mechanics (i.e. activation-dependent force response of muscles to length and velocity transients), and thereby predicting *in vivo* forces, remains challenging, particularly for fast and perturbed conditions (Daley and Biewener, 2011; Dick et al., 2017; Dickinson et al., 2000; Wakeling et al., 2021). These challenges persist in part because the current paradigms of muscle function – the sliding filament and the swinging cross-bridge theories (Hill, 1922; Montesano et al., 2020) – are commonly represented by ‘Hill-type’ muscle models that predict force depending on quasi-static isometric force–length and isotonic force–velocity relationships (Ahn, 2012; Blümel et al., 2012; Dickinson et al., 2000; Sponberg et al., 2023).

The defining parameters of Hill-type models include series elasticity, isometric (passive and active) force–length and isotonic force–velocity relationships (Ahn, 2012; Blümel et al., 2012; Holt and Azizi, 2016). While these parameters are useful as standardized measurements for comparing contractile properties among different muscles and treatments, they do not consistently capture the intrinsic mechanical effects that time-varying loads impose on muscle force and work under the dynamic conditions that characterize *in vivo* movements (Biewener and Daley, 2007; Libby et al., 2019; McGowan et al., 2013; Sponberg et al., 2011, 2023). *In vivo* load transitions, associated with length and velocity transients, result in history-dependent variation in muscle force, work and power (Ahn et al., 2006; Biewener and Daley, 2007; Daley and Biewener, 2003; Edman and Josephson, 2007; Josephson, 1985; Robertson and Sawicki, 2015), especially in terrestrial legged locomotion. To understand the contributions of intrinsic muscle properties to *in vivo* force production, elucidation of muscle's dynamic force–length and force–velocity relationships is required.

The classic work loop technique has demonstrated the contributions of intrinsic muscle properties by controlling stimulation and length trajectories in *ex vivo* and *in situ* preparations while measuring force and work output over a contraction cycle (Ahn, 2012; Josephson, 1985; Robertson and Sawicki, 2015). When force is plotted against length change over a cycle, the area enclosed within the loop is the muscle work output (Ahn, 2012). Previously, sinusoidal and saw-tooth contractions have been used to characterize how muscle mechanical output varies with cycle frequency, activation phase and other factors (e.g. Askew and Marsh, 1997; Askew et al., 2001; Rome and Lindstedt, 1998; Tu and Dickinson, 1994), which emulates *in vivo* length changes that occur during certain types of movement such as flying, swimming and chewing. Additionally, a work loop technique has been employed using *ex vivo* mouse muscles with estimated length trajectories from an OpenSim model as inputs to investigate stimulation intensity and its role during force production of the human soleus (Bukovec et al., 2020). Work loops have also been used to investigate the interactions between foot shape, external loads and muscle force and work output in swimming biological systems using advance bio-robotics techniques (Richards, 2011; Richards and Clemente, 2012). These studies highlight the versatile mechanical function of muscle, and the complex relationship between intrinsic muscle properties, length trajectory, activation and work output (Ahn et al., 2006; Ahn and Full, 2002; Bukovec et al., 2020; Richards, 2011; Richards and Clemente, 2012; Richards and Eberhard, 2020; Robertson and Sawicki, 2015). However, in contrast to the more continuously cyclical dynamics of swimming, flying and chewing, legged locomotion involves sudden transitions between unloaded and loaded states, which cannot be accurately replicated by sinusoidal length trajectories (Richards and Biewener, 2007; Sponberg et al., 2023). Work loops generated from direct *in vivo* measurements in legged locomotion have revealed that muscles exhibit complex strain trajectories in natural movements, allowing them to act like springs, struts, motors and/or brakes, depending on the context (e.g. Daley and Biewener, 2003; Roberts et al., 1997). Additionally, *in vivo* perturbation studies have revealed that work loop shape and work output are especially sensitive to the strain transients that occur with abrupt changes in applied loads (Daley and Biewener, 2003, 2011; Daley et al., 2009; Gordon et al., 2020; Schwaner et al., 2023). The purpose of this study was to expand a key gap in our understanding of muscle function between quasi-static *ex vivo* experiments and dynamic *in vivo* locomotion.

Here, we employed an ‘avatar’ work loop method (Bemis and Nishikawa, 2023; Rice, 2020; Rice et al., 2023), where appropriately scaled length trajectories from muscle fascicles, as measured *in vivo*, are imposed *ex vivo* on an experimentally accessible and well-characterized muscle preparation: mouse extensor digitorum longus (EDL) (Askew and Marsh, 1997; Charles et al., 2016; Hämäläinen and Pette, 1993; James et al., 1995). Muscles were scaled to physiologically appropriate lengths using the optimal length of maximum isometric force production of mouse EDL (Shelley et al., 2024). To better understand the dynamics of muscle mechanics during *in vivo* locomotion, we aimed to: (1) replicate force production under *ex vivo* conditions using *in vivo* length trajectories from speed-varying tasks; and (2) compare force and work output to sinusoidal trajectories at equivalent frequencies, to gain insight into how strain transients contribute to force production at varying gaits.

We hypothesized that non-linear intrinsic muscle properties, including dynamic muscle responses due to transients in strain and velocity, strongly influence time-varying force and work output of muscle, in addition to activation. Strain transients can elicit dynamic, time-varying changes in force output in cyclical contractions, even without changes in activation (Daley and Biewener, 2011; Libby et al., 2019; Sponberg et al., 2023). By contrast, traditional force–length and force–velocity relationships do not capture dynamic responses, and traditional work loop techniques that use sinusoidal length changes at *in vivo* frequencies fail to reproduce the complex length transients that are typical of *in vivo* movement and therefore critical for understanding *in vivo* muscle force and work. We propose the muscle ‘avatar’ as a new standardized approach for characterizing fundamental dynamic muscle function across several species, as it deploys realistic length trajectories under controlled conditions.

## MATERIALS AND METHODS

### Muscle preparation

EDL muscles from adult male and female wild-type mice (*Mus musculus*, B6C3Fe a/a-Ttn, *n*=8) before considered geriatric (60–250 days of age) (Hagan, 2017) were used in this study. A colony was established at Northern Arizona University (NAU) from breeder mice obtained from the Jackson Laboratory (Bar Harbor, ME, USA). Mice were fed *ad libitum* and euthanized just prior to muscle extraction. The Institutional Animal Care and Use Committee (IACUC) at NAU approved the use of the animals and experimental protocol (#21-001).

The largest head of the EDL complex (which is composed of 9 muscles) was removed surgically (Bemis and Nishikawa, 2023) with 4-0 silk sutures tied in square knots at the distal and proximal muscle–tendon junctions just prior to *ex vivo* experiments. Extracted EDL muscles were attached to a dual-mode muscle lever system (Series 300B, Aurora Scientific, Inc., Aurora, ON, Canada). During experiments, muscles were submerged in an aerated bath of Krebs–Henseleit solution containing (in mmol 1^−1^): NaCl (118); KCl (4.75); MgSO_4_ (1.18); KH_2_PO_4_ (1.18); CaCl_2_ (2.54); Hepes (11.5); and glucose (10.0), at 21°C and 7.4 pH. The bath was aerated with a 95% O_2_ and 5% CO_2_ gas mixture. While submerged, muscles were suspended between two platinum electrodes that delivered 1 ms square-wave stimuli from a Grass S88 stimulator. Before finding optimal length (*L*_0_) of maximum isometric force, a series of 80 V, 180 Hz conditioning twitches was applied to the muscle until twitch force reached a steady state (Hakim et al., 2013).

To find *L*_0_, muscles were stimulated tetanically at supramaximal stimulation (80 V, 180 Hz, 500 ms). Submaximal stimulation (45 V, 110 Hz; ∼80% of maximum isometric force during supramaximal stimulation) was used during all experimental protocols to more closely emulate *in vivo* activation (Manuel et al., 2019; Tijs et al., 2021; Wakeling et al., 2021). Isometric force was measured at *L*_0_ using submaximal stimulation for 500 ms before and after the experimental protocol. Except for twitch stimuli, muscles were rested for 3 min between trials. If muscles lost more than 10% of their submaximal isometric force at *L*_0_, they were considered fatigued or damaged and were not included in the analysis. No muscles had to be excluded in the present study. After experimental trials, muscles were removed from the rig, sutures and excess tendon were removed, muscles were patted dry and weighed to the nearest microgram to determine physiological cross-sectional area (PCSA). PCSA was calculated using the formula: muscle mass (g)/(*L*_0_×1.06 g cm^−3^) (Rice et al., 2023). Pennation angle of the mouse EDL is small (12 deg); the difference between the muscle and fascicle length was also small (< 3%) and assumed to be very similar.

### Using *in vivo* medial gastrocnemius parameters in *ex vivo* EDL work loops

**Fig. 1.**
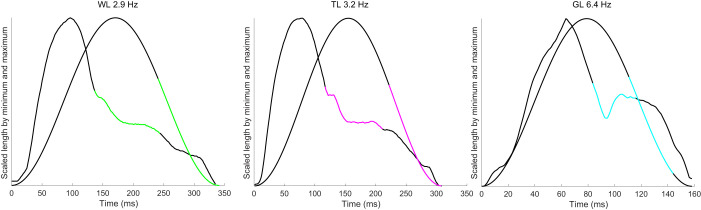
**Scaled ‘avatar’ and sinusoidal length trajectories for different gaits against time.** Adjusted stimulation is not shown because of severe overlap in EMG-based and adjusted onset and duration periods. Foot contact is closely correlated to EMG activation in all three ‘avatar’ strides. (A) Scaled ‘avatar’ walk length trajectory and sinusoidal length trajectory at the same frequency (WL 2.9 Hz). Green line shows onset and duration of EMG-based stimulation. (B) Scaled ‘avatar’ trot length trajectory and sinusoidal length trajectory at the same frequency (TL 3.2 Hz). Magenta line shows onset and duration of EMG-based stimulation. (C) Scaled ‘avatar’ gallop length trajectory and sinusoidal length trajectory at the same frequency (GL 6.4 Hz). Blue line shows onset and duration of EMG-based stimulation.

In a previous study (Wakeling et al., 2021), muscle fascicle length trajectories, activation and force were measured *in vivo* from rat medial gastrocnemius (MG) using sonomicrometry, electromyography and leaf-spring tendon buckles during walking, trotting and galloping on a level, uphill and downhill treadmill. Hindlimb gait kinematics and stride frequencies were determined using video motion capture (Wakeling et al., 2021) (see Fig. S1). *In vivo* fascicle length trajectories (Fig. 1) obtained from a single rat (Rat #4 of Wakeling et al., 2021) on a level treadmill at varying stride frequencies [walk (WL) 2.9 Hz, trot (TL) 3.2 Hz and gallop (GL) 6.4 Hz] were used in this study to define length trajectories and control stimulation timing of EDL muscles during *ex vivo* work loop experiments.

**Fig. 2.**
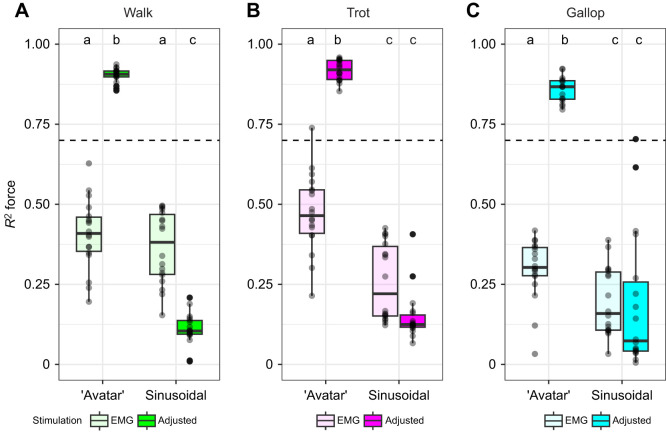
**‘Avatar’ length trajectories and adjusted stimulation most accurately predicted *in vivo* force.** Comparison of *R*^2^ between scaled *in vivo* and *ex vivo* time-varying force values for all gaits (A, walk; B, trot; and C, gallop), length trajectories (‘avatar’ versus sinusoidal) and stimulation patterns [electromyography (EMG)-based versus adjusted stimulation protocol]. Letters indicate statistical groupings across gait (walk, trot and gallop) from the ANOVA. Box plots show 25th, 50th (median) and 75th percentile; whiskers are 1.5× interquartile range. Outliers are indicated by circles. Dashed lines indicate the threshold for successfully replicating time-varying force.

To investigate the contributions of intrinsic properties and rapid length transients on muscle forces during stretch–shortening cycles (SSCs) with varying frequencies, we used one representative stride each from walking (Figs 1A and 2A; WL), trotting (Figs 1B and 2B; TL) and galloping (Figs 1C and 2C; GL) in work loop experiments on mouse EDL. *In vivo­* length trajectories (hereafter referred to as ‘avatar’ length trajectories) used for EDL *ex vivo* work loops were matched to the *in vivo* frequencies of the MG (Fig. 2). Sinusoidal length trajectories with no rapid length transients at the same amplitudes and frequencies were also used to compare time-varying force and work output. For each length trajectory (‘avatar’ and sinusoidal), SSCs were performed twice in each condition (24 total work loops per muscle).

Although rat MG and mouse EDL have similar muscle architecture (Charles et al., 2016; Eng et al., 2019), they have different pennation angles, MG pennation angle being twice that of EDL (Tijs et al., 2021). Additionally, rat MG and mouse EDL have different operating length ranges (Table 1), and different activation and deactivation kinetics (Hämäläinen and Pette, 1993; Manuel et al., 2019). Therefore, adjustments to work loop parameters were necessary. Parameters adjusted included length (starting length and total excursion) and stimulation (intensity, onset and duration). For this and previous studies (Rice, 2020; Rice et al., 2023), muscle starting length and total excursion were adjusted (Shelley et al., 2024) to match the observed pattern of passive tension rise during *in vivo* SSCs (see Bemis and Nishikawa, 2023, for methodology). Stimulation onset and duration were manually adjusted to match the timing of the rise and duration of active tension (Bemis and Nishikawa, 2023). Stimulation intensity was standardized at submaximal (∼80% of maximum isometric force) to best emulate *in vivo* EDL activation (Manuel et al., 2019). Preliminary studies using ‘avatar’ length trajectories for all three gaits were performed on EDL at different starting lengths (i.e. −10% *L*_0_, −5% *L*_0_, *L*_0_ and +5% *L*_0_) and total excursions (i.e. 5% *L*_0_, 10% *L*_0_ and 15% *L*_0_). Optimized starting lengths varied among gaits (walk +5% *L*_0_, trot *L*_0_ and gallop −5% *L*_0_) whereas an excursion of 10% *L*_0_ best matched the passive tension rise observed *in vivo* for all gaits.

**Table 1.**
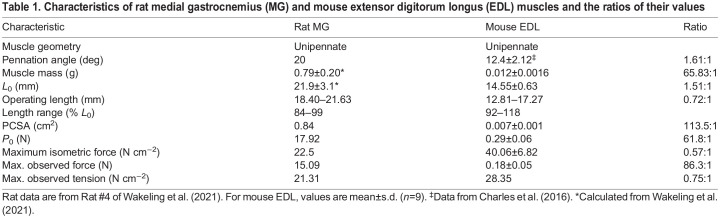
Characteristics of rat medial gastrocnemius (MG) and mouse extensor digitorum longus (EDL) muscles and the ratios of their values

Two stimulation patterns were used in our work loop experiments, electromyography (EMG) based and adjusted (Table 2). The first pattern (EMG based) was based on measured EMG activation of the MG, which typically started just before or at foot contact (Eng et al., 2019; Wakeling et al., 2021). EMG activation onset was defined as the first occurrence of EMG intensity increasing above the baseline by two standard deviations (Roberts and Gabaldón, 2008; Tenan et al., 2017). Stimulation duration was calculated using the time of last observed EMG activation change of two standard deviations and subtracting the measured onset. We accounted for the difference between *in vivo* excitation–contraction coupling (ECC) delay in rats (∼25 ms; Schmid et al., 2019) and the much shorter ECC delay during *ex vivo* work loop experiments due to direct electrical stimulation (∼5 ms) by stimulating the EDL 20 ms later than observed for *in vivo* activation. Each gait condition incorporated its unique EMG-based stimulation onset and duration (Table 2).

**Table 2.**
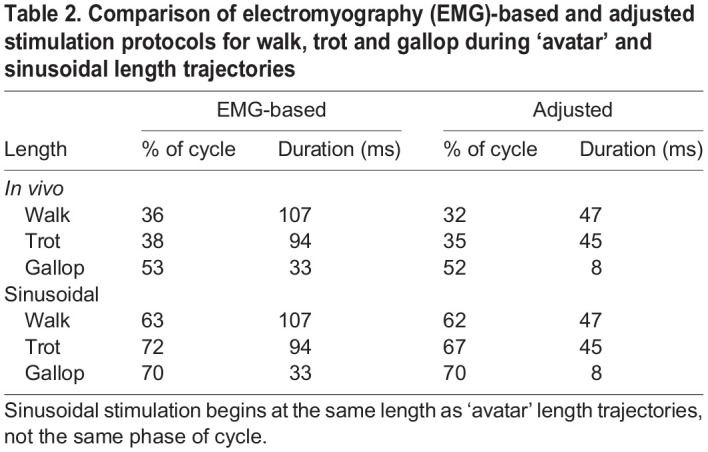
Comparison of electromyography (EMG)-based and adjusted stimulation protocols for walk, trot and gallop during ‘avatar’ and sinusoidal length trajectories

The second stimulation pattern (adjusted) was also based on matching *in vivo* passive and active time-varying force. Stimulation onset and duration were adjusted manually to best match MG force production *in vivo* using ‘avatar’ length trajectories. Once stimulation adjustment was manually achieved for specific *in vivo* length trajectories during all gaits, stimulation onset was calculated to start at the same length as ‘avatar’ length trajectories for the same duration of time in sinusoidal conditions (Fig. 2). We hypothesized the need to manually adjust stimulation arises mostly from differences in activation–deactivation kinetics between rat MG and mouse EDL (Abbate et al., 2002; Eng et al., 2008; Hämäläinen and Pette, 1993). Adjusted stimulus onset was earlier for walk and trot but was the same as the EMG-based stimulation pattern for gallop (Table 2). Stimulation duration was always shorter for the adjusted stimulation pattern as compared with the EMG-based stimulation pattern, specifically by 44% for walk, 48% for trot and 25% for gallop (Table 2), and thus all gaits had a unique adjusted stimulation pattern (Table 2).

All six length trajectories (‘avatar’ and sinusoidal for all three gaits) were tested using both stimulation patterns (EMG based and adjusted) with submaximal stimulation at their respective starting lengths (+5% *L*_0_ at walk, *L*_0_ for trot and −5% *L*_0_ for gallop), and total excursion (10% of *L*_0_). A total of 12 conditions were performed on each muscle, including all combinations of six length trajectories and two stimulation patterns with two replicates for each experiment, for a total *N*=216 SSCs in the dataset. Length trajectories and stimulation patterns were performed in randomized order for each muscle. All reported length measurements in this study are relative length. Relative length was calculated using each individual muscle's measured *L*_0_ (*L*/*L*_0_) (Biewener and Patek, 2018).

There were also several work loop variables that were measured to compare dynamic muscle function in the differing experimental conditions. These include (1) highest shortening and (2) average velocity (m s^−1^), which was calculated over the entire SSC. Also, (3) relative peak force (% *P*_0_) and (4) length at peak force (% *L*_0_) were calculated. In the custom LabView (National Instruments, Austin, TX, USA) program used to collect data, length was a controlled input and force was an unconstrained output (see Bemis and Nishikawa, 2023, for more details). Only the phase of the cycle with active force was used in the reported measurements of this study. To compare force of rat and mouse muscles, both rat and mouse forces were scaled by the maximum isometric force (*F*_0_) using custom MatLab code (MATLAB, R2021a). From length and force, (5) net work was calculated across the entire SSC. Net work was calculated relative to muscle mass (mJ g^−1^). Finally, (6) minimum and maximum active velocity of the length trajectories from stimulation onset to peak force were calculated to compare velocity transients.

### Statistical analysis

We hypothesized that using ‘avatar’ length trajectories would improve experimental matching to *in vivo* muscle force compared with sinusoidal length trajectories at the same stride frequency, as a result of length transients from applied force in the *in vivo* length trajectories. We computed the coefficient of determination (*R*^2^) and root mean squared error (RMSE) for comparisons between observed time-varying force of MG (1 rat muscle) and EDL (9 mouse muscles) to measure the similarity (*R*^2^) and total error (RMSE)*.* Force of MG, EDL ‘avatar’ and sinusoidal length trajectories at equivalent amplitude and frequency were also compared using *R*^2^ and RMSE. All data points within a single cycle (walk *n*=1358, trot *n*=1242, gallop *n*=634) were used to calculate *R*^2^ and RMSE values for both force and length. RMSE was highly correlated with *R*^2^ (−0.84) and is therefore not reported. The hypothesis was rejected if ‘avatar’ *R*^2^≤sinusoidal *R*^2^ between MG and EDL forces*.* Optimization of length and stimulation parameters was considered valid if adjusted stimulation *R*^2^>EMG-based *R*^2^ for ‘avatar’ length trajectories across gaits. Gait (WL, TL, GL) and stimulation (EMG based, adjusted) were combined within the ‘stimulation’ effect because of their inherent non-independence (see Table 2). To account for clustering associated with repeated measures within each muscle, we used linear mixed effects models with muscle as a random factor, and *R*^2^ of time-varying force production as the response variable (Muhammad, 2023). The variance component of the random effect of ‘muscle’ was consistently small (see Table S1), suggesting minimal clustering by muscle preparation. Analysis of variance (ANOVA) was used on the linear mixed effects models to test the hypotheses that fixed effects of (1) length (‘avatar’ versus sinusoidal; fixed), (2) stimulation (WL EMG based, WL adjusted, TL EMG based, TL adjusted, GL EMG based and GL adjusted; fixed) and (3) the interaction length×stimulation influenced the similarity (*R*^2^) and total error (RMSE) when replicating time-varying force (RStudio 2020; https://posit.co/products/open-source/rstudio/). A type III ANOVA with Satterthwaite's method was used to test for statistical significance of fixed effects. Tukey's honestly significance difference (HSD) was used for *post hoc* pairwise comparisons to determine which specific groups' means were significantly different from each other. We used Cohen's effect size (*d*) to compare effect sizes between variables. Alpha was considered at *P*<0.05 for statistical analyses including ANOVA and *post hoc* comparisons.

To test the validity of the ‘avatar’ method for investigating work output of MG using EDL muscles, we compared work (mJ g^−1^) for all gaits, length trajectories and stimulation protocols. The mean work of all *in vivo* strides for Rat #4 during walk, trot and gallop was calculated and compared with that of EDL using two-sided Student's *t*-tests. All means are reported with standard deviation (mean±s.d.). Cohen's effect size (*d*) was used to analyze the magnitude of differences in means. Effect sizes were considered small (*d*<0.2), medium (*d*<0.5) or large (*d*>0.8) (Lakens, 2013). We hypothesized that the ‘avatar’ with adjusted stimulation would result in similar means (*P*>0.05) of work in EDL and MG for all three gaits with small effect sizes. Small effect sizes would indicate large overlap of rat MG and mouse EDL populations. Additionally, we performed an ANOVA on a linear mixed model constructed with net work per cycle as the response variable with the same fixed (length, stimulation, length×stimulation) and random effects (muscle) accounting for repeated measures, described earlier. Work loop variables were only compared within mouse muscles and not across the rat MG.

The additional measured work loop variables – (1) highest shortening velocity (m s^−1^); (2) average velocity (m s^−1^); (3) relative peak force (% *P*_0_); (4) length at peak force (% *L*_0_); and (6) minimum and maximum active velocity – were compared across ‘avatar’ and sinusoidal length trajectories and gait frequencies. These linear mixed models and all *post hoc* comparisons were constructed with the same fixed and random effects as described above for *R*^2^ and net work per cycle. For all gaits, data were visually inspected to confirm that distributions were not grossly different from normal. ANOVA is generally considered robust to analyze data with moderate deviations from normality (Blanca et al., 2017). All test statistics for ANOVA performed are reported in Table S2.

To determine the relationships among independent (length, gaits, stimulation protocols) and dependent work loop variables, a principal component analysis (PCA) was performed in RStudio 2020 (https://posit.co/products/open-source/rstudio/) using ‘avatar’ and sinusoidal length trajectories. All independent variables and dependent variables were included except greatest shortening velocity as it was partially correlated with average velocity (0.38). Prior to preforming PCA, the data were standardized to ensure each variable had a mean of zero and a standard deviation of one, preventing variables with differing scales from dominating the analysis. The first two principal components, which accounted for the majority of the variance, were analyzed further with an ANOVA to interpret the underlying structure of the data. Results of PCAs were graphed in clusters with 95% confidence intervals demarcated to allow more concise visualization.

## RESULTS

### Comparison of rat MG and mouse EDL muscles

Fascicle length changes of the proximal and distal rat MG (Wakeling et al., 2021) were averaged to estimate muscle belly length *in vivo* (Wakeling et al., 2021), whereas total muscle length was measured for the EDL muscles. While MG fascicle length changes are not directly comparable to the EDL length measured, the ‘avatar’ method aims to capture the time-varying pattern of change in length, which was similar at all locations in the rat MG (walk *R*^2^=0.99; trot *R*^2^=0.99; gallop *R*^2^=0.99). The EDL and MG are both unipennate muscles (Table 1). Pennation angle of the mouse EDL is small (12 deg), so the difference between muscle length and fascicle length is also small (<3%; Table 1), which may explain why MG fascicle length changes were comparable to EDL length changes. PCSA differed between mouse EDL and rat MG by a factor of 113.5 (mean±s.d. EDL 0.007±0.001 cm^2^, MG 0.84 cm^2^). Lengths of EDL and MG overlapped during walk, trot and gallop, with EDL ranging from 14.98 to 18.48 mm and MG ranging from 18.40 to 21.63 mm (Table 1). *L*_0_ in MG (21.9 mm) was ∼1.5 times longer than EDL (14.55 mm). Peak isometric forces observed differed by a factor of 86 (EDL 0.18 N, MG 15.09 N). EDL produced around 2 times more isometric force (41 N cm^−2^) than MG (22 N cm^−2^).

### Comparison of *in vivo* and ‘avatar’ work loops

#### Evaluations based on *R*^2^ and RMSE

**Fig. 3.**
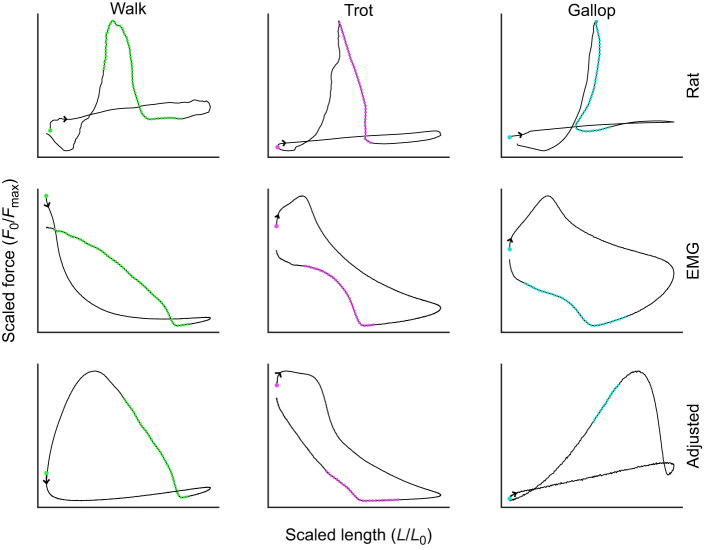
**Representative work loops of rat and sinusoidal length trajectories for walk (green), trot (magenta) and gallop (blue).** Circle indicates the beginning of the cycle for the muscle. Arrows show direction if the beginning and end of the cycle occur at the same length and force. Colors indicate stimulation onset and duration.

**Fig. 4.**
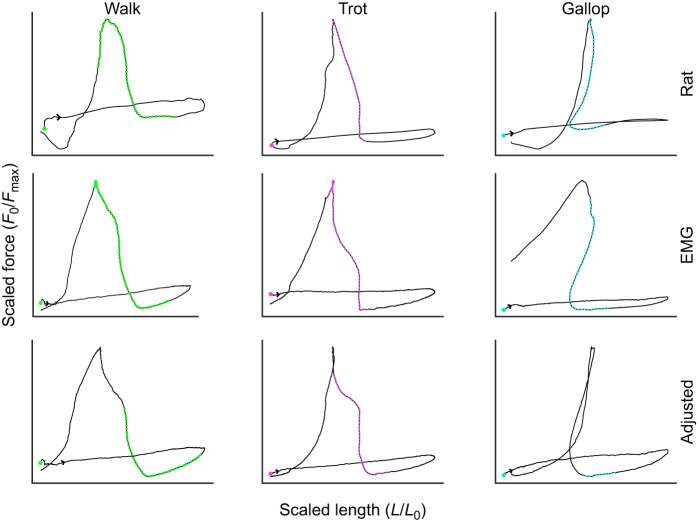
**Representative work loops of rat and ‘avatar’ length trajectories for walk (green), trot (magenta) and gallop (blue).** Circle indicates the beginning of the cycle for the muscle. Arrows show direction if the beginning and end of the cycle occur at the same length and force. Colors indicate stimulation onset and duration.

EDL muscles exhibited variation in force–length behavior among length trajectories and stimulation protocols (Figs 2–4). For walk, *R*^2^ values between MG and EDL ‘avatar’ time-varying forces ranged from 20% to 63% for EMG-based stimulation, and from 86% to 94% for adjusted stimulation (Fig. 2A), whereas *R*^2^ values for sinusoidal trajectories ranged from 15% to 50% for EMG-based stimulation, and from 1% to 21% for adjusted stimulation (Fig. 2A). For trot, *R*^2^ values between MG and EDL ‘avatar’ trajectories ranged from 21% to 74% for EMG-based stimulation, and from 85% to 96% for adjusted stimulation (Fig. 2B), whereas *R*^2^ values for sinusoidal trajectories ranged from 12% to 42% for EMG-based stimulation, and from 7% to 41% for adjusted stimulation (Fig. 2B). For gallop, *R*^2^ values between MG and EDL ‘avatar’ trajectories ranged from 3% to 42% for EMG-based stimulation, and from 80% to 92% for adjusted stimulation (Fig. 2C), whereas *R*^2^ values using sinusoidal trajectories ranged from 3% to 39% for EMG-based stimulation and from 1% to 71% for adjusted stimulation (Fig. 2C).

Sinusoidal length trajectories differed in shape from ‘avatar’ length trajectories for all gaits (walk mean *R*^2^=0.17; trot mean *R*^2^=0.08; and gallop mean *R*^2^=0.68; see Figs 3 and 4). Sinusoidal length trajectories did not display the distinguishing shape characteristics of both EDL ‘avatar’ and MG work loops, lacking the partitioning into swing and stance phases and having a more rectangular shape, as observed in previous studies (Rice, 2020; Rice et al., 2023). The proportion of variance in MG force explained by sinusoidal length trajectories (*R*^2^) varied greatly among gaits and stimulation protocols (Fig. 2) but they never replicated *in vivo* MG force as accurately as, or with similar overall error to, ‘avatar’ length for both stimulation protocols (see Fig. 2 for mean *R*^2^ values).

The *R*^2^ values for time-varying force varied by length (ANOVA, *P*<0.0001, *d*=2.23), stimulation (*P*<0.0001, *d*=0.95) and their interaction (*P*<0.0001, *d*=1.63). ‘Avatar’ length trajectories were more influenced by changes in stimulation than were sinusoidal length trajectories, whereas *R*^2^ values of sinusoidal length trajectories were statistically similar to each other (Tukey's HSD, *P*>0.05) (Fig. 2). Adjusted stimulation had a larger positive effect on *R*^2^ among ‘avatar’ length trajectories than among sinusoidal trajectories (Fig. 2C). This is not surprising given adjusted stimulation was implemented to improve *ex vivo* time-varying force predictions using the ‘avatar’ length trajectories.

#### Evaluations based on net work per cycle

**Fig. 5.**
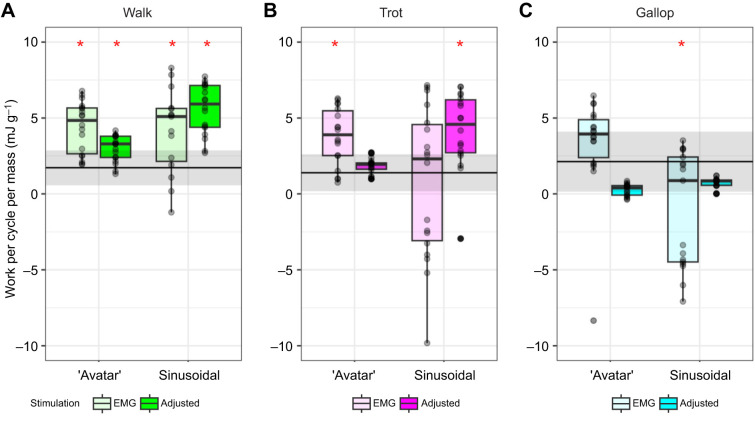
**‘Avatar’ trajectories and adjusted stimulation best reproduced net work output per cycle of the *in vivo* rat medial gastrocnemius (MG).** Horizontal black line indicates average net work and shading indicates standard deviation for *in vivo* work of rat MG. Jitter box plots show work per cycle for *ex vivo* mouse EDL for all gaits (A, walk; B, trot; C, gallop), lengths (‘avatar’ and sinusoidal) and stimulation patterns (EMG-based and adjusted stimulation protocol). Samples that differed statistically from *in vivo* rat MG are indicated by red asterisks. Box plots show 25th, 50th (median) and 75th percentiles; whiskers are 1.5× interquartile range. Outliers are indicated by circles.

Net work output per cycle varied depending on stimulation pattern (ANOVA, *P*<0.0001, *d*=0.009) and the interaction of length×stimulation (*P*<0.0001, *d*=0.49), but did not differ between length trajectories (‘avatar’ versus sinusoidal; *P*=0.89, *d*=0.75). *Post hoc* comparisons indicated that ‘avatar’ lengths varied more among gaits (WL, TL, GL) than between stimulation patterns (EMG-based versus adjusted), while sinusoidal length trajectories varied more between stimulation patterns than among gaits (Fig. 5). This is not surprising given that sinusoidal length trajectories at the varying gaits did not have any transients present that differentiated them from each other.

‘Avatar’ and sinusoidal EDL length trajectories did not reproduce *in vivo* work of MG consistently (Fig. 5). *Ex vivo* experiments, despite the length and stimulation protocol, produced more work than during *in vivo* locomotion, except in gallop ‘avatar’ adjusted and sinusoidal with both stimulation protocols*.* Sinusoidal length trajectories with EMG-based stimulation produced a more variable amount of work than any other condition for all gaits (Fig. 5). ‘Avatar’ length trajectories for walk with both stimulation protocols, and sinusoidal length trajectories with adjusted stimulation produced significantly more work per cycle than rat MG (Student's *t*-test, ‘avatar’ adjusted *P*<0.02, *d*=1.28; EMG-based *P*=0.05, *d*=1.80; Student's *t*-test, sinusoidal adjusted *P*=0.01, *d*=2.61). For trot, ‘avatar’ length trajectory with adjusted stimulation and sinusoidal with EMG-based stimulation were similar to *in vivo* work. For gallop, sinusoidal length trajectories with adjusted stimulation produced significantly less work than rat MG (Student's *t*-test, *P*=0.0007, *d*=1.32). All other length and stimulation protocols during gallop produced similar work per cycle to *in vivo* rat MG. Only during gallop did *ex vivo* experimental protocols (‘avatar’ length with adjusted stimulation and sinusoidal length with both stimulations) produce less work than rat MG (Fig. 5C).

### Comparison of work loop variables among *ex vivo* length and stimulation protocols

Length trajectories varied greatly depending on gait, especially for gallop (see Fig. 1). Sinusoidal length trajectories for all gaits had average velocities close to zero. A fundamental difference between sinusoidal and ‘avatar’ length trajectories was that the latter had abrupt changes in length rate (i.e. length and velocity transients) and timing of peak length that were not present in sinusoidal trajectories (see Fig. 1). These abrupt length and velocity transients in MG length trajectories corresponded to the timing of foot contact (Wakeling et al., 2021). MG typically reached its maximum shortening velocity immediately before foot contact and its maximum stretch velocity shortly after, especially during gallop.

### Highest and average velocity

Highest and average velocities (m s^−1^) were positively correlated with each other (0.38). Highest shortening velocity varied with length (ANOVA, *P*<0.0001, *d*=3.9); stimulation (highest velocity *P*=0.44, *d*=0.05) and their interaction (highest velocity *P*=0.55, *d*=0.14) were not significant in the linear model built. Highest shortening velocity increased with gait for both ‘avatar’ and sinusoidal trajectories, which is not surprising given the differences in the frequencies at which they occur (WL 2.8 Hz, TL 3.2 Hz, GL 6.8 Hz). EMG-based and adjusted stimulation produced the highest shortening velocity for gallop (mean EMG 0.08±0.02 m s^−1^, adjusted 0.09±0.02 m s^−1^). *Post hoc* comparisons showed that walk and trot produced similar highest shortening velocities. Average velocity varied with length (ANOVA, *P*=0.82, *d*=0.91); stimulation (*P*=0.82, *d*=0.02) and their interaction (*P*=0.99, *d*=0.02) were not significant in the linear model. Only ‘avatar’ length trajectories during gallop were significantly greater than other length and stimulation conditions. The larger average velocities are due to the large transients present in ‘avatar’ gallop length trajectories associated with foot contact, which are not present in the other length trajectories (Fig. 1C). Gallop ‘avatar’ length trajectories with both stimulation patterns produced higher average velocity (mean EMG 1.37±1.35 m s^−1^, adjusted 1.39±1.39 m s^−1^) than any other length–stimulation combinations.

### Relative peak force

Relative peak force (% *P*_0_) varied with length (ANOVA, *P*<0.0001, *d*=2.3), stimulation (*P*<0.0001, *d*=0.95) and their interaction (*P*<0.0001, *d*=1.6). For gallop, ‘avatar’ and sinusoidal trajectories produced higher relative peak force during EMG-based stimulations (mean EMG-based 0.46±0.064% *P*_0_; adjusted 0.25±0.125% *P*_0_). Adjusted stimulations during gallop were only 8 ms long (Table 2). Additionally, ‘avatar’ gallop trajectory with adjusted stimulation produced less relative peak force than ‘avatar’ trajectories during trot with EMG-based (mean 0.56±0.22% *P*_0_) and adjusted (mean 0.47±0.169% *P*_0_) stimulation and walk with EMG-based (mean 0.54±0.201% *P*_0_) and adjusted (mean 0.44±0.151% *P*_0_) stimulation. Adjusted stimulations across all length trajectories were less than EMG-based simulations (Table 2).

### Muscle length at peak force

Muscle length at peak force (% *L*_0_) varied with length (ANOVA, *P*<0.0001, *d*=3.4), stimulation (*P*<0.0001, *d*=0.5) and their interaction (*P*<0.0001, *d*=0.5). This result is unsurprising, given that walk, trot and gallop had unique starting lengths (+5% *L*_0_, *L*_0_ and −5% *L*_0_) and peak lengths (+15% *L*_0_, +10% *L*_0_, +5% *L*_0_). All combinations of length and stimulation conditions differed significantly in muscle length at peak force, except length trajectories from the same gait with differing stimulation protocols (walk EMG-based versus adjusted stimulation; trot EMG-based versus adjusted stimulation; gallop EMG-based versus adjusted stimulation). The EMG-based and adjusted stimulation protocols had equivalent mean length at peak force within each gait (walk 1.09±0.01% *L*_0_; trot 1.04±0.0151% *L*_0_; gallop 1.00±0.01% *L*_0_).

### Minimum and maximum active velocity

**Fig. 6.**
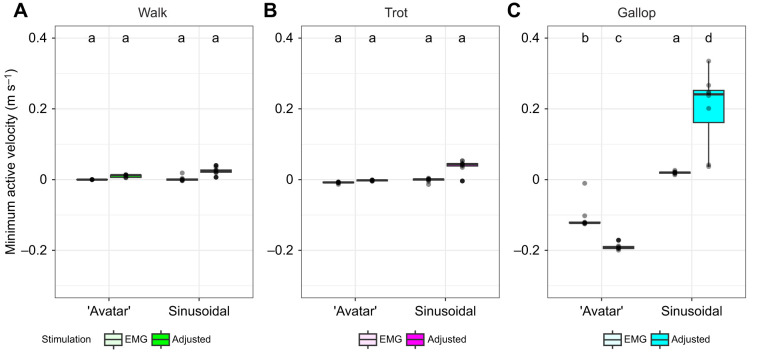
**Minimum active velocity (from stimulation onset to peak force) for ‘avatar’ and sinusoidal length trajectories for the different gaits.** Comparison of length trajectories for both stimulation patterns (EMG based and adjusted) across gaits (A, walk; B, trot; and C, gallop), length trajectories and stimulations. Letters indicate statistical groupings across all gaits from the ANOVA. Box plots show 25th, 50th (median) and 75th percentile; whiskers are 1.5× interquartile range. Outliers are indicated by circles.

**Fig. 7.**
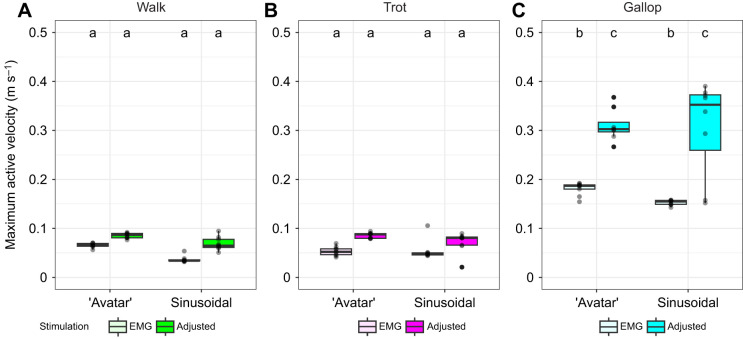
**Maximum active velocity (from stimulation onset to peak force) for ‘avatar’ and sinusoidal length trajectories for the different gaits.** Comparison of length trajectories for both stimulation patterns (EMG based and adjusted) across gaits (A, walk; B, trot; and C, gallop), length trajectories and stimulations. Letters indicate statistical groupings across all gaits from the ANOVA. Box plots show 25th, 50th (median) and 75th percentile; whiskers are 1.5× interquartile range. Outliers are indicated by circles.

Minimum active velocity of length (m s^−1^) varied by length, stimulation and their interaction (ANOVA, *P*<0.0001). The largest effect of minimum velocity of length was length (*d*=2.3), which is to be expected given the differences in shortening during each length trajectory (Fig. 1). Minimum active velocity was produced similarly between all walk and trot length and stimulation protocols (Fig. 6). All ‘avatar’ length trajectories of walk and trot were nearly sinusoidal with near-constant concentric contractions during stimulation onset to peak force. Maximum active velocity (m s^−1^) varied by length, stimulation and their interaction (ANOVA, *P*<0.0001). The largest effect on maximum active velocity was length (*d*=2.3) as a result of the difference in shortening velocity and the eccentric portion of ‘avatar’ length trajectory, which is not present in the sinusoidal trajectory during gallop (Figs 1C and 7). This variation is most likely attributed to the differences in stimulation protocols (Table 1).

### PCA

**Fig. 8.**
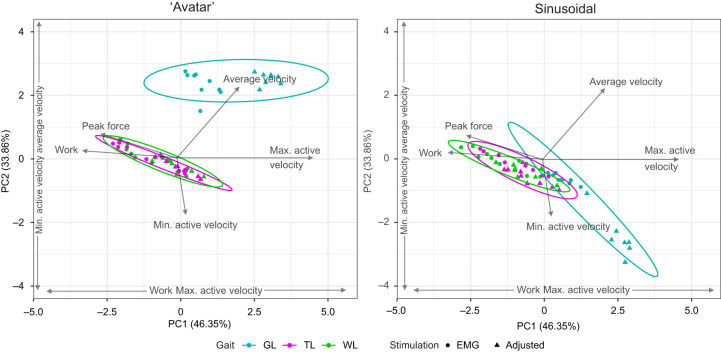
**Principal component analysis (PCA) of all variables categorized by fixed effects (EMG-based and adjusted stimulation) using ‘avatar’ and sinusoidal length trajectories during *ex vivo* experiments.** Variables were peak force (% *P*_0_), average velocity (mm s^−1^), work per cycle (mJ g^−1^) and minimum and maximum active velocity (mm s^−1^). The first two principal component axes (PC1 and PC2) explain 80% of the variance. PC1 positive loadings are minimum (loadings=0.02) and maximum (0.58) active velocities, and average velocity (0.33). Work (−0.55) and peak force (−0.51) are negative loadings on PC1. PC2 consists of average velocity (0.64), peak force (0.26) and work (0.11). Negative loadings on PC2 are minimum (−0.44) and maximum (–0.003) stimulation length velocity. Gallop is most distinguished in both ‘avatar’ and sinusoidal length trajectories on PC1 and PC2.

To better parse out contributions of work loop variables to force production and work output, we performed a PCA using normalized peak force (% *P*_0_), average velocity (m s^−1^), work per cycle (mJ g^−1^) and stimulated lengths minimum and maximum velocity (mm s^−1^) of EDL for all three gaits (WL, TL and GL), both stimulation protocols (EMG-based and adjusted) and both length trajectories (sinusoidal and ‘avatar’) (Fig. 8). Principal component 1 and 2 (PC1, PC2) explain 80% of the variance present in the data. The highest positive driver of PC1 was maximum active velocity (0.58) and the highest negative driver was work (−0.55). On PC1, gaits were distinguished by length, stimulation and their interaction (ANOVA, *P*<0.01). Variation between length trajectories was not clearly defined on PC1 except during gallop (Fig. 8). Additionally, in both ‘avatar’ and sinusoidal length trajectories, stimulation protocols were distinguished (Fig. 8). These differences were influenced by work output and maximum active velocity because of overall time SSCs were stimulated (Table 1).

Variation on PC2 was most positively affected by average velocity (0.64) and most negatively by minimum active velocity (−0.72). On PC2, length, stimulation and their interactions (ANOVA, *P*<0.0001) were distinguished. Most distinct along PC2 was the differentiation between gallop and the other gaits, in both ‘avatar’ and sinusoidal length trajectories (Fig. 8). During gallop, sinusoidal length trajectories were distinguished by stimulation protocols while ‘avatar’ length trajectories were not (Fig. 8).

## DISCUSSION

The innovation of the ‘avatar’ method for studying *in vivo* muscle mechanics is to use a widely studied and readily available muscle (i.e. mouse EDL) in controlled *ex vivo* experiments to represent the *in vivo* force length dynamics from a distinctly different muscle. The ultimate usefulness of the ‘avatar’ method is dependent on how accurately a convenient, inexpensive, readily available and well-characterized laboratory rodent model can be used for this goal.

Rice (2020) and Rice et al. (2023) previously used mouse EDL to represent the *in vivo* performance of guinea fowl lateral gastrocnemius (LG) during treadmill running over obstacles (Rice, 2020; Rice et al., 2023). They found that length, stimulation and interactions between length and stimulation had large effects on work loop variables, including work per cycle and peak force, and that the length×stimulation interaction had the largest effects on work loop variables. They also found that work loops using ‘avatar’ length trajectories from *in vivo* level and obstacle strides more accurately predicted *in vivo* LG forces than sinusoidal work loops, even with the stimulation pattern being consistent across conditions. The results of the present study confirm and extend Rice et al.’s (2023) results. In this study, we used ‘avatar’ trajectories from rat MG during walking, trotting and galloping on a level treadmill. Methodological differences between Rice et al. (2023) and the present study are minor, but important. Experimental parameters that differed from Rice et al. (2023) included matching *in vivo* frequencies of individual gaits, using EMG-based as well as adjusted stimulation protocols for each individual gait, and systematic variation of starting length to approximate *in vivo* passive force for each gait. Compared with the results from this previous ‘avatar’ study, our protocol changes resulted in more consistent predictions (*R*^2^>0.70) of *in vivo* force in work loop experiments.

Similar to Rice and colleagues (2023), we found that work loops using ‘avatar’ length trajectories from *in vivo* level and obstacle strides predicted *in vivo* MG forces more accurately than sinusoidal work loops. We also found that length transients, stimulation and interactions between length transients and stimulation had significant effects on work and force output, especially as speed increases. When muscles increase their shortening velocity they undergo larger length and velocity transients from applied loads (i.e. foot contact; Fig. 2C), which may be a strategy for economy (Roberts et al., 1997) and stability to have load-mediated responses (Biewener and Daley, 2007; Daley and Biewener, 2006; Daley et al., 2006). This is most clearly demonstrated by gallop being distinguished from the ‘avatar’ length trajectories and sinusoidal length trajectories in our PCA (Fig. 8). ‘Avatar’ gallop underwent the most extreme length and velocity transients after foot contact with the substrate (Fig. 2C). This finding is consistent with the emerging view that muscle is a composite material that actuates movement by developing force in response to combined effects of activation-dependent viscoelastic properties, and the time-varying and load-dependent resistance of active muscle to deformation by applied loads (Nishikawa, 2020; Tahir et al., 2018). The results also demonstrate that the *in vivo* fascicle length trajectory of a muscle represents its response to applied forces, including the muscle's internal forces that change with activation, forces applied by antagonistic muscles, force transmission from segmentally linked muscles across joints, and reaction forces from the environment, as they occur during foot contact during legged locomotion (Rice, 2020; Rice et al., 2023). The results also highlight that the activation/deactivation kinetics substantially influence cyclical force production in addition to the muscle's force–velocity (Hahn et al., 2023; Sugi and Ohno, 2019), force–length (Holt and Azizi, 2014; Marsh, 1999) and operating length (Shelley et al., 2024) properties.

Legged locomotion involves non-steady perturbations to muscle length that arise from interaction with the substrate, which sinusoidal length trajectories do not capture (see Fig. 2). Applied loads from foot contact with the substrate affect muscle fascicle length and length rate in ways that are not as apparent during steady sinusoidal-like movements, such as unperturbed flight, chewing or swimming (Richards and Biewener, 2007; Tobalske, 2007). Traditional work loop techniques (Ahn, 2012; Josephson, 1985, 1999), which control length and stimulation of *ex vivo* muscles at specified frequencies and timing, have provided key insights into muscle work and power production during steady locomotion (Dickinson et al., 2000). Purely sinusoidal length trajectories, typically used in work loops, lack variation in length rate that results from variable loading during locomotion (Rice et al., 2023; Sponberg et al., 2023). This study, in conjunction with other recent studies implementing the ‘avatar’ approach (Bemis and Nishikawa, 2023; Rice, 2020; Rice et al., 2023), demonstrates the contribution of length and velocity transients to muscle force during dynamic legged locomotion.

There was a significant influence of SSC frequency across gaits on ‘avatar’ work loop variables (peak force, muscle length at peak force, average velocity, highest shortening velocity, maximum and minimum active velocity, and work per cycle). For most measured variables, including average and highest shortening velocity, walk and trot did not differ significantly between stimulation conditions. The only variable that differed was muscle length at peak force, likely because of differences in the initial lengths that were used to best replicate *in vivo* rat MG passive force. In contrast, ‘avatar’ gallop with both stimulation patterns differed from the other gaits, suggesting that effective stiffness of the mouse EDL was reduced during gallop compared with walk and trot. This reduction in effective stiffness can be attributed to higher shortening velocities that prevent cross-bridge attachments (Fenwick et al., 2017) and the differences in stimulation duration. Peak force and work per cycle were lowest during the gallop length trajectories. This is not surprising, given that gallop length trajectories began at shorter lengths, were stimulated for the shortest amount of time and had significantly higher velocity than the other gaits. Manually adjusted starting lengths and amplitudes do not correspond with observed *in vivo* MG length trajectories (Wakeling et al., 2021) and work output (Fig. 5). When manually adjusting starting and maximum lengths, we aimed to best replicate the *in vivo* passive forces rather than the *in vivo* strain magnitudes. The need to use different starting lengths and strain amplitudes to replicate similar passive forces might relate to differences in muscle architecture, intrinsic stiffness and operating length ranges between mouse EDL and rat MG. Further investigation is needed to address whether the operating length ranges of a given muscle are constant or whether additional parameters (i.e. stimulation intensities, maximum length) can be manipulated in conjunction with varying length ranges to accurately replicate *in vivo* force production. Investigation into these potential combinations of ‘avatar’ input parameters when attempting to accurately replicate *in vivo* time-varying force could provide insights into the dynamic nature of muscle function and its adaptability under different physiological conditions.

After stimulation adjustment, *ex vivo* mouse EDL muscles consistently reproduced *in vivo* forces of the rat MG at all frequencies (see Fig. 4). Adjusted stimulation yielded higher *R*^2^ (80–92%) and lower RMSE values for all gaits than EMG-based stimulation, and *R*^2^ values were similar across gaits. We found high similarity in the patterns of time-varying force despite differences in muscle properties, as well as differences between *in vivo* and *ex vivo* preparations. For example, the PCSA of MG from Rat #4 (0.841 cm^2^) was ∼96 times greater than that for EDL (mean 0.00867 cm^2^), and the peak force observed for a length amplitude of 10% *L*_0_ in the mouse EDL was 28.35 N cm^−2^ versus 21.31 N cm^−2^ for a length amplitude of 15% *L*_0_ in rat MG (Table 1). This finding suggests that the mouse EDL is stiffer than the rat MG (Table 1), potentially explaining why adjustment of stimulation onset was required and yielded marked improvements in *R*^2^ compared with the EMG-based stimulation protocol.

In addition to altering stimulation onset, the simulation adjustment protocol also reduced the EDL stimulation duration by >25% in all gaits compared with *in vivo* EMG duration in the MG (Table 2). There may be several explanations as to why the reduced stimulation duration in ‘avatar’ EDL more accurately replicated *in vivo* force of rat MG, including differences in activation and deactivation dynamics and twitch kinetics between these muscles. Rat MG is composed of slower fiber types than mouse EDL (Abbate et al., 2002; Eng et al., 2008; Manuel et al., 2019). Optimal operating frequencies of mouse EDL and rat MG also differ (Hämäläinen and Pette, 1993; Manuel et al., 2019). Rat MG galloping frequency on average was ∼7 Hz while the mouse EDL operates at ∼10 Hz during galloping (James et al., 1995). Non-physiological reasons for the reduction in stimulation duration may include the EMG signal-to-noise ratio or the EMG electrodes detecting movement artifacts and/or cross-talk from surrounding muscles (Reaz et al., 2006). More studies using a variety of readily available laboratory muscles with different fiber types are needed to understand how individual muscle properties affect stimulation timing at different SSC frequencies during dynamic movements.

In this study, optimization of length and stimulation parameters was unique for each length trajectory. All length trajectories during all gaits had a length excursion of 10% *L*_0_, but individual gaits had varying maximum (walk +15% *L*_0_; trot +10% *L*_0_; gallop +5% *L*_0_) and starting lengths (walk +5% *L*_0_; trot *L*_0_; gallop −5% *L*_0_). We suggest that operating lengths that differ between the MG and EDL muscles may play a role in determining optimal starting and maximum lengths (Bemis and Nishikawa, 2023; Shelley et al., 2024). For all gaits except gallop, the adjusted stimulation protocol effectively removed the ECC delay that was included in the EMG-based stimulation protocol. We believe that this likely occurred because ECC delays are typically measured under isometric conditions, but work loop experiments are non-isometric. Previous studies have shown that length changes affect the rate of force development during muscle activation (Sandercock and Heckman, 1997; Shue and Crago, 1998). Thus, our finding indicates that isometric ECC coupling delay may not be physiologically relevant during non-isometric cyclical movements.

Some previous work loop studies investigated muscle–tendon unit length changes because muscle–tendon interactions are thought to increase energy efficiency during locomotion (Roberts, 2002; Roberts et al., 1997). Both studies found that energy exchange between muscles and tendons facilitated economical force production during a wide variety of tasks in turkeys. While these fundamental studies demonstrate how muscle–tendon units actuate movement to power whole-body and joint mechanics, the results are not directly relevant for this study. We did not measure changes in tendon lengths or joint torques during *ex vivo* experiments, and the scope of the projects is different. Our study asked how applied loads affect individual muscle forces given observed *in vivo* length trajectories and activation, whereas previous studies focused on how whole-body and joint mechanics can be inferred from muscle–tendon unit lengths, activation and force output.

### Future directions and limitations

In this study, *in vivo* source and *ex vivo* target muscle architecture were relatively similar, whereas muscle size, fiber-type composition and *in vivo* function differed substantially. It would be informative in future experiments to compare additional muscle types and species with varying size and muscle architecture, function and fiber type composition to investigate the how differences in intrinsic muscle properties influence the interaction between strain, activation and work output. For example, comparison of results from *ex vivo* mouse EDL with *in situ* mouse MG experiments will enable quantification of the relative contribution of muscle size, fiber-type composition, activation and deactivation kinetics, and series elastic compliance to muscle force and work.

Here, we used fascicle strain as input into the muscle ergometer to control whole muscle–tendon unit length change and demonstrated the ability to accurately replicated *in vivo* time-varying force production despite the simplifying assumption of negligible in-series compliance and fiber-gearing effects. The ability to replicate whole muscle–tendon contraction dynamics based upon fascicle strain as input may be due to the small pennation angle and removal of the series elastic component (tendon) of the mouse EDL. Muscles with complex architecture (i.e. larger tendon to muscle ratios, larger pennation angles) could require different inputs, such as accurately modelled muscle–tendon length changes based on joint kinematics to accurately replicate *in vivo* time-varying force production. Here, we focused on investigating the effects of strain transients on muscle function, because strain transients arise from abrupt changes in loading that are particularly relevant to terrestrial locomotion. Related previous work (Richards, 2011; Richards and Clemente, 2012; Robertson and Sawicki, 2015) has focused on using biologically realistic loads as inputs to the ergometer, measuring fascicle strain and work as output. However, these studies have generally focused on movements that involve sinusoidal load and strain patterns (i.e. swimming) without abrupt changes in load associated with substrate contact. Replicating biologically realistic loads during legged locomotion is difficult compared with swimming because of the challenges of simulating realistic contact dynamics with the environment. This limitation likely contributes to the difficulty in accurately replicating *in vivo* net work output per cycle in our *ex vivo* conditions.

Our ‘avatar’ work loops did not consistently replicate *in vivo* work per cycle (see Fig. 4). Most *ex vivo* length trajectory inputs (‘avatar’ as well as sinusoidal) across all gaits and stimulation protocols yielded statistically more work per cycle than the rat MG *in vivo*. Only a few frequencies and length–stimulation patterns were similar to rat MG in mean values (*P*>0.05) (Fig. 3). This result is likely due to differences in how muscles are stimulated between *in vivo* and *ex vivo* experiments, rather than the accuracy of the ‘avatar’ method. Submaximal square-wave (1 ms) stimulation was used in this study; however, *in vivo* muscle activation is more complex, with time-varying intensities from recruitment of different fiber types (Lee et al., 2011), compared with whole-field electrical stimulation (Bukovec et al., 2020). Future experiments that use time-varying stimulation patterns to better emulate *in vivo* activation patterns observed from EMG would be useful to further expand our understanding of muscle force and work during *in vivo* locomotion. Another limiting factor may be that stimulation of muscles during *ex vivo* experiments requires higher intensities (80% of supramaximal stimulation) to elicit a tetanic response from the muscles than what is observed *in vivo.* More direct measurements and comparison are needed between *ex vivo* and *in vivo* stimulation intensities to further investigate that limitation.

### Conclusions

This study demonstrates: (1) that using trajectories derived from *in vivo* measurements improves prediction of individual stride variability in muscle force for different gaits and stride frequencies; and (2) that sinusoidal length trajectories do not emulate *in vivo* force production during terrestrial locomotion as a result of only being stimulated during the concentric portion of the SSC unlike *in vivo* muscle (Fig. 2). The results of this study also indicate that dynamic strain effects help to regulate force and regulate work output of muscle (Josephson, 1999; Sponberg et al., 2023), and that muscle force and work are highly influenced by the frequency of the SSC (Robertson and Sawicki, 2015) during non-sinusoidal SSCs (Bemis and Nishikawa, 2023; Rice et al., 2023). An emerging new perspective considers muscles not just as motors that produce force in response to activation but also as actively tunable materials in which activation changes the material properties and force arises through resistance to deformation (Nguyen and Venkadesan, 2021 preprint; Nishikawa, 2020). The results of this study support the view that muscle forces arise from resistance to deformation by applied loads, which are represented by length and velocity transients seen in muscle fascicles (see Fig. 1).

Establishing relationships between length and velocity transients, frequency and stimulation patterns during *in vivo* force production and work modulation allows us to understand the fundamental properties of muscle across different species, architecture and activation/deactivation kinetics. Additionally, using *in vivo* measured strain trajectories allows us to investigate how abrupt changes in load influence muscle function without the need to develop complex simulations that involve difficult to replicate contact transitions (between unloaded and loaded states). By further investigating these core characteristics, we can develop models that accurately predict *in vivo* muscle forces, even in cases where direct measurements are unavailable. This study aimed to advance our knowledge of muscle function and develop more reliable methodology for cross-species comparisons while developing more robust predictive capabilities in biomechanics.

## Supplementary Material

10.1242/jexbio.248177_sup1 Supplementary information
