# Supplementary material for: Investigating in vivo force and work production of rat medial gastrocnemius at varying locomotor speeds using a muscle avatar
Source: J Exp Biol. 2024 Nov 13;227(22):jeb248177. doi: 10.1242/jeb.248177 (PMC11586523; doi:10.1242/jeb.248177)
Supplement: Supplementary information [file jexbio-227-248177-s1.pdf]

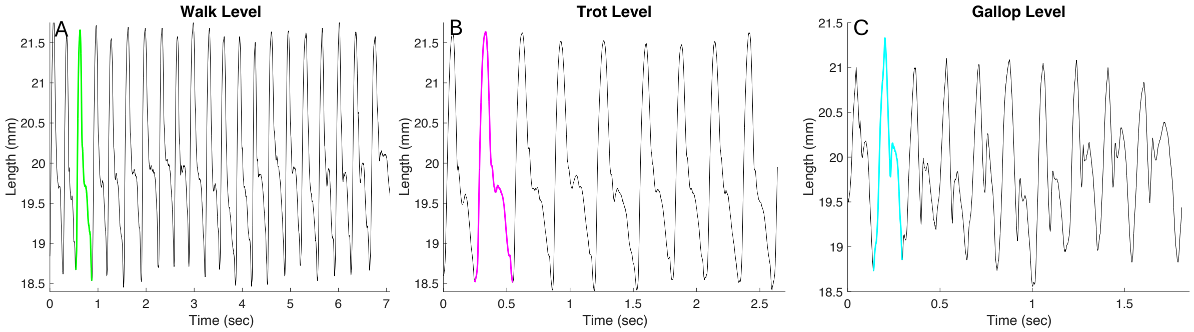

Fig. S1. Total trial of rat MG showing selected representative strides during A) walk, B) trot, and C) gallop. A) Selected stride during walk (green). B) Selected stride during trot (magenta). C) Selected stride during gallop (blue). Data from Wakeling et al. (2021). These colors will be used throughout the article.

Table S1. Reported variance and standard deviation (S.D.) of random factor 'muscle' in the full linear mixed-effect model.

Response	Random effect variance	Random effect S.D.	Residual variance	Residuals S.D.
Net work per cycle	0.86	0.93	6.19	2.49
Highest velocity	6.82e-07	0.0008	4.72e-05	0.007
Average velocity	0.00	0.00	0.30	0.55
Relative peak force	0.20	0.14	0.007	0.09
Length at peak force	0.0001	0.01	0.0002	0.01
Min. active velocity	3.42e-05	0.006	1.32e-03	0.04
Max. active velocity	0.0001	0.01	0.002	0.04

Journal of Experimental Biology • Supplementary information

Table S2. Test statistics of all ANOVA performed on the on fixed effects in the linear mixed effects model. SS = sum of squares, Mean sq = mean square, N. DF = numerator degrees of freedom, D. DF = denominator degrees of freedom, Act = activation.

Variable	SS	Mean Sq	N. DF	D. DF	F-value	p-value
**R^2 of time-varying force**						
Length	10.31	2.06	5	193.99	192.59	3.3e-73
Activation	1.89	1.89	1	194		
Length * act						
**Net work / cycle**						
Length	674.4	134.9	5	194	21.8	<2.2e-16
Activation	0.12	0.12	1	194	0.82	0.89
Length * act	284.6	56.92	5	194	9.19	7.2e-08
**Highest shortening velocity**						
Length	0.14	0.03	5	194	593.41	<2e-16
Activation	2e-05	2e-05	1	194	0.64	0.44
Length * act	1e-04	3e-05	5	194	0.81	0.55
**Average vel**						
Length	51.03	10.21	5	202	33.65	<2e-16
Activation	0.02	0.02	1	202	0.05	0.82
Length * act	0.02	4e-04	5	202	0.01	0.99
**Relative peak force**						
Length	1.73	0.34	5	194	46.28	<2.2e-16
Activation	1.31	1.31	1	194	174.84	<2.2e-16
Length * act	0.24	0.05	5	194	6.5	1.2e-05
**Length at peak force**						
Length	0.33	0.07	5	194	469.7	<2.2e-16
Activation	0.007	0.007	1	194	48.5	4.9e-11
Length * act	0.006	0.001	5	194	9.78	1.5e-07
**Min. active velocity**						
Length	1.33	0.27	5	194	199.75	<2.2e-16
Activation	0.06	0.06	1	194	45.12	2e-09
Length * act	0.41	0.08	5	194	61.24	<2.2e-16
**Max. active velocity**						
Length	1.53	0.31	5	194	198	<2.2e-16
Activation	0.25	0.25	1	194	163.8	<2.2e-16
Length * act	0.21	0.04	5	194	27.7	<2.2e-16

Journal of Experimental Biology • Supplementary information
